# Sodium Nitroprusside as a Xenobiotic Model of Oxidative and Nitrosative Stress in Cellular and Zebrafish Systems

**DOI:** 10.3390/jox16010029

**Published:** 2026-02-06

**Authors:** Carlos Alberto-Silva, Felipe Assumpção da Cunha e Silva, Brenda Rufino da Silva, Leticia Ribeiro de Barros, Adolfo Luis Almeida Maleski, Maricilia Silva Costa

**Affiliations:** 1 Experimental Morphophysiology Laboratory, Natural and Humanities Sciences Center (CCNH), Universidade Federal do ABC (UFABC), São Bernardo do Campo 09606-070, SP, Brazil; fhellcunha@gmail.com (F.A.d.C.e.S.); brendarufino33@gmail.com (B.R.d.S.); leticia_ribeiro@hotmail.com (L.R.d.B.); adolfomaleski@gmail.com (A.L.A.M.); 2 Instituto de Pesquisa e Desenvolvimento—IP&D, Universidade do Vale do Paraíba—UNIVAP, Av. Shishima Hifumi, 2911, São José Dos Campos 12244-390, SP, Brazil; mscosta@univap.br

**Keywords:** xenobiotic-induced toxicity, nitrosative stress, reactive oxygen species (ROS), neurotoxicity, behavioral phenotyping, neurodegenerative disorders

## Abstract

Oxidative and nitrosative stress are central mechanisms in the pathogenesis of neurodegenerative diseases, where excessive production of reactive oxygen and nitrogen species (ROS/RNS) leads to mitochondrial dysfunction, membrane damage, and neuronal death. In this study, we established and compared short-term (2 h) and long-term (20 h) exposure paradigms to sodium nitroprusside (SNP), used as a xenobiotic nitric oxide donor, in two neuronal cell lines (mHippoE-18 and PC12) and zebrafish larvae, aiming to provide a preclinical framework for neurodegenerative drug discovery. In vitro, SNP exposure caused concentration-dependent reductions in viability and alterations in oxidative balance, with mHippoE-18 cells exhibiting higher susceptibility than PC12 cells. In the short-term exposure paradigm, cytotoxicity was primarily associated with membrane disruption at higher concentrations, whereas oxidative stress contributed more strongly at intermediate doses. In the long-term exposure, mHippoE-18 cells showed strong integrated correlations between ROS, LDH release, and viability loss, highlighting their increased vulnerability to nitrosative stress. In zebrafish, SNP exposure impaired metabolic activity and swimming behavior in both paradigms. Long-term exposure led to consistent dose-dependent increases in ROS, accompanied by locomotor deficits tightly linked to energy metabolism. Overall, the higher sensitivity of mHippoE-18 cells compared with PC12 cells, together with the dose-dependent metabolic and behavioral impairments observed in zebrafish, indicates that cellular responses partially mirror in vivo outcomes. This integrative approach underscores the value of combining neuronal cell lines with zebrafish larvae to capture complementary aspects of SNP-induced neurotoxicity and to strengthen preclinical evaluation of candidate compounds with protective or therapeutic potential. These findings support the use of SNP as a xenobiotic model to probe nitrosative stress-driven neurotoxicity across cellular and organismal systems.

## 1. Introduction

Oxidative and nitrosative stress are key contributors to neurodegeneration, affecting neuronal integrity through damage to lipids, proteins, and DNA mediated by reactive oxygen and nitrogen species (ROS/RNS) [1]. They have been consistently associated with the pathogenesis of major neurodegenerative diseases, including Alzheimer’s disease, Parkinson’s disease, Huntington’s disease, and amyotrophic lateral sclerosis [2]. In these conditions, excessive ROS/RNS generation overwhelms endogenous antioxidant defenses, leading to mitochondrial dysfunction, excitotoxicity, and activation of apoptotic pathways [2]. These processes not only accelerate neuronal death but also contribute to selective neuronal vulnerability, thereby explaining the progressive and region-specific nature of neurodegeneration [3].

Experimental evidence from cellular systems demonstrates that oxidative stress acts as an early driver of neurodegenerative cascades rather than a secondary by-product of neuronal loss [4], while complementary in vivo studies in zebrafish confirm that oxidative imbalance produces both structural and behavioral phenotypes reminiscent of human disease [5]. Given these pathophysiological mechanisms, modeling oxidative and nitrosative stress in vitro and in vivo has become an essential strategy for drug discovery [6,7,8]. Experimental paradigms that reproduce toxic insults and subsequent recovery phases allow researchers to evaluate both protective and therapeutic interventions under controlled conditions. For instance, zebrafish and neuronal cell lines have been widely used to simulate oxidative injury, most commonly through exposure to hydrogen peroxide (H_2_O_2_), thereby providing valuable insights into redox imbalance, behavioral alterations, and the efficacy of candidate molecules [4,7]. However, despite its popularity, repeated H_2_O_2_ application may lead to inconsistent cellular responses and limited reproducibility, reducing its utility as a robust experimental paradigm [9].

To address these limitations, sodium nitroprusside (SNP), a stable nitric oxide (NO) donor widely used as a xenobiotic model, has emerged as an alternative approach, since it induces both oxidative and nitrosative stress, thereby mimicking more complex and physiologically relevant conditions of neuronal damage [10,11,12]. As an exogenous xenobiotic, SNP enables controlled induction of nitrosative stress, allowing the systematic evaluation of toxicodynamic responses across different biological systems. In the classic PC12 model, high concentrations of SNP (e.g., 400 μmol·L^−1^) trigger apoptotic cascades through activation of stress kinases, p53 stabilization, caspase activation, Bcl-2 down-regulation, and increased ROS, alongside DNA damage and impaired antioxidant defenses [13,14,15,16,17]. Beyond mechanistic insights, SNP-induced neurotoxicity in PC12 cells has also served as a platform to evaluate neuroprotective strategies. Pre-treatment with low, subtoxic SNP doses (~100 μmol·L^−1^) can precondition PC12 cells and partially mitigate apoptosis induced by subsequent high-dose SNP, via attenuation of stress signaling and reduction in apoptotic markers [18]. Similarly, diverse protective agents, such as a huperzine A derivative (M3) [16], artemisinin (a well-known anti-malaria drug) [17], phytochemical compounds [19], 1,2,4-triazole derivatives [20] or selol (an organic selenite triglyceride) [21], have been reported to reduce SNP-induced ROS, enhance anti-apoptotic signaling, and increase cell survival in PC12 cultures.

To date, most in vitro studies employing SNP in neuronal models focus on protective pre-treatment paradigms, with only limited examples of co-treatment-based rescue strategies. For instance, selol co-administered with SNP attenuates ROS generation and apoptosis in neuronal cell lines, supporting translational relevance for therapeutic intervention [21]. In contrast, true post-treatment approaches, in which a therapeutic agent is applied after the SNP insult, remain largely unexplored. Nevertheless, reports with H_2_O_2_-based models suggest that such paradigms are feasible. The flavonoid didymin, for example, was administered after H_2_O_2_-induced injury, a process known as neurorescue, resulting in increased cell viability, reduced ROS, and modulation of key antioxidant and apoptotic markers in SH-SY5Y neuronal cells [22]. Conversely, post-treatment with curcumin in PC12 cells following H_2_O_2_ exposure was ineffective, highlighting the complexity of evaluating therapeutic versus protective interventions [23].

In this context, the present study aimed to establish and characterize short-term and long-term exposure models of SNP-induced oxidative and nitrosative stress, using this compound as a xenobiotic probe in neuronal systems. By integrating in vitro assays in mHippo-E18 and PC12 cells with in vivo analyses in zebrafish larvae, we sought to compare cellular susceptibility, redox imbalance, and behavioral outcomes under distinct paradigms of toxic exposure and recovery. Importantly, PC12 cells, derived from rat pheochromocytoma, represent a classical and widely used neuronal model, while mHippo-E18 cells originate from embryonic mouse hippocampus and provide a complementary system that more closely reflects hippocampal vulnerability implicated in memory-related neurodegenerative disorders. These combined approaches not only provide novel insights into cell type-specific responses to SNP but also generate a robust experimental framework to evaluate the efficacy of protective and therapeutic interventions. Ultimately, this work proposes a translationally relevant platform for the bioprospecting of candidate compounds against oxidative stress-related neurodegenerative processes.

## 2. Materials and Methods

### 2.1. Reagents

All reagents employed in the experiments were of analytical grade (purity ≥ 98%) and sourced from established commercial suppliers, including Gibco BRL (New York, NY, USA), Sigma-Aldrich (St. Louis, MO, USA), and Synth (Diadema, SP, Brazil). Stock solutions were prepared in ultrapure deionized water passed through a 0.22 μm membrane filter and exhibiting a resistivity greater than 18.2 MΩ (Millipore, Burlington, MA, USA). Cell culture media and related supplements were obtained from Thermo Fisher Scientific (Waltham, MA, USA). Sodium nitroprusside (SNP) was dissolved in sterile water immediately prior to each experiment to minimize degradation caused by light exposure.

### 2.2. Cell Lines and Maintenance

Two neuronal cell lines were employed: mouse embryonic hippocampal mHippoE-18 cells (CLU199, CELLutions Biosystems, Toronto, ON, Canada) and rat pheochromocytoma PC12 cells (CRL-1721™, ATCC, Manassas, VA, USA). The mHippoE-18 cells were cultured in high-glucose Dulbecco’s Modified Eagle Medium (DMEM; DH10) supplemented with 10% fetal bovine serum (FBS), penicillin (100 U·mL^−1^), streptomycin (100 μg·mL^−1^), and amphotericin B (25 μg·mL^−1^). PC12 cells were maintained in low-glucose DMEM (D10) under the same supplementation conditions. All culture media were supplemented with heat-inactivated fetal bovine serum, obtained by incubation at 56 °C for 30 min. Cells were maintained at 37 °C under humidified conditions in an atmosphere containing 5% CO_2_ and 95% air, using a Forma™ Series 3 Water Jacketed CO_2_ incubator (Thermo Scientific Inc., MA, USA). Culture media were renewed every 2 to 3 days, and subculturing was performed when cells reached approximately 80% confluence. PC12 cells were detached using 0.05% trypsin–EDTA, whereas mHippoE-18 cells were passaged using Versene solution composed of NaCl (140 mmol·L^−1^), KCl (2.7 mmol·L^−1^), Na_2_HPO_4_ (10 mmol·L^−1^), KH_2_PO_4_ (1.8 mmol·L^−1^), and EDTA (0.5 mmol·L^−1^).

### 2.3. SNP-Induced Oxidative Stress Effects In Vitro

SNP-induced oxidative stress was assessed in both neuronal cell models. mHippoE-18 and PC12 cells were plated at densities of 2.5 × 10^3^ and 5 × 10^3^ cells per well, respectively, in 96-well plates (Nest Biotechnology, Rahway, NJ, USA) containing a final volume of 100 μL and allowed to attach for 24 h prior to treatment. Subsequently, cells were subjected to short-term and long-term exposure models.

#### 2.3.1. Experimental Design In Vitro

**Figure 1:**
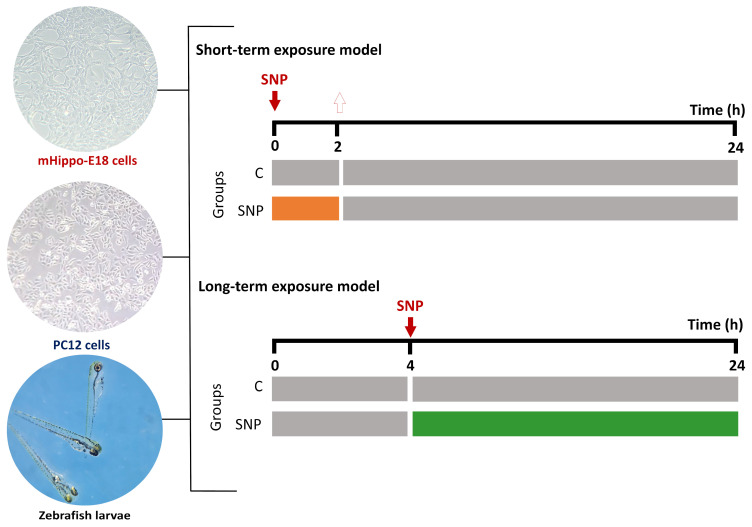
Schematic representation of the experimental designs for the short-term and long-term exposure models using mHippoE-18 cells, PC12 cells, and zebrafish larvae. In the short-term exposure, cells or larvae were treated with SNP for 2 h, followed by replacement with SNP-free medium or water for an additional 22 h before endpoint measurements. In the long-term exposure, cells or larvae were pre-incubated in culture medium or water for 4 h, after which SNP was added and maintained for 20 h before analysis. Control groups (C) received identical handling but without SNP exposure. Representative micrographs of mHippoE-18 cells (100×), PC12 cells (100×), and zebrafish larvae (25×) are shown on the left.

To investigate the cytotoxic effects of SNP, two experimental protocols were performed (Figure 1). In the short-term exposure, cells were treated with SNP diluted in DH10 or D10 medium (0.1, 0.5, and 1 mmol·L^−1^) for 2 h. Following exposure, the medium was replaced with fresh, SNP-free medium, and cells were incubated for an additional 22 h at 37 °C with 5% CO_2_. In the long-term exposure, cells were pre-incubated in DH10 or D10 medium alone for 4 h, after which the medium was replaced with fresh medium containing SNP (0.1, 0.5, and 1 mmol·L^−1^) and maintained for 20 h at 37 °C with 5% CO_2_. Untreated cells were included as the control group in both models. After treatments, oxidative stress induced by SNP was assessed by crystal violet staining for cell viability [24], lactate dehydrogenase (LDH) release as a marker of membrane integrity [25], and 2′,7′-dichlorodihydrofluorescein diacetate (H_2_DCF-DA; Sigma–Aldrich, St. Louis, MO, USA) staining for ROS production [26].

#### 2.3.2. Cell Viability by Crystal Violet Staining and LDH Release Assays

Following the experimental exposures, culture supernatants were collected and the adherent cells were stained with a 0.5% (*w*·*v*^−1^) crystal violet solution. Absorbance was subsequently recorded at 570 nm using a BioTek Epoch microplate spectrophotometer (BioTek, Santa Clara, CA, USA), in accordance with previously established protocols [24]. LDH activity was quantified using a kinetic UV method based on pyruvate/NADH, following the manufacturer’s instructions (Katal Biotecnológica, Belo Horizonte, Brazil). Briefly, samples (20 μL) were incubated with the working reagent containing Tris buffer (100 mmol·L^−1^, pH 7.5), sodium pyruvate (0.6 mmol·L^−1^), and NADH (0.25 mmol·L^−1^). The reduction in absorbance at 340 nm was continuously monitored at 37 °C, and lactate dehydrogenase activity was calculated and reported as units per liter (U·L^−1^). Results were summarized as box-and-whisker plots derived from three independent experiments, each conducted in sextuplicate.

#### 2.3.3. ROS Assay

Aliquots of culture medium (50 μL) from each experimental condition were transferred to black 96-well plates (SPL Life Sciences, Pocheon-si, Republic of Korea). Subsequently, 145 μL of PBS and 5 μL of H_2_DCF-DA (1 mmol·L^−1^; Sigma–Aldrich^®^, St. Louis, MO, USA) were added to each well. The plates were incubated at 37 °C for 1.5 h in the absence of light. Fluorescence signals were recorded using a multimode microplate reader (BioTek Synergy HT, Santa Clara, CA, USA) with excitation and emission wavelengths set at 480 and 530 nm, respectively. Data were normalized to the control group and presented as box-and-whisker plots obtained from three independent experiments, each performed in sextuplicate.

### 2.4. SNP-Induced Oxidative Stress Effects In Vivo

#### 2.4.1. Zebrafish Maintenance, Husbandry, and Egg Collection

Adult wild-type zebrafish (WT) were housed in the zebrafish facility of the Experimental Morphology Laboratory at the Federal University of ABC (UFABC) under standardized environmental conditions, including a constant water temperature of 28 °C and a controlled 14/10 h light–dark cycle. Fish were housed in glass aquaria filled with distilled water supplemented with sodium chloride (60 μg·mL^−1^; pH 7.0). All animal procedures were conducted in accordance with the European Directive 2010/63/EU [27] and the guidelines established by the Brazilian National Council for the Control of Animal Experimentation (CONCEA) [28]. Adult zebrafish were fed commercial dry food twice daily and supplemented with Artemia nauplii on the day preceding mating. Male and female fish were maintained at a 1:1 ratio. For embryo production, spawning devices were placed in the tanks on the evening prior to breeding. Following fertilization, eggs were collected and transferred to E3 embryo medium composed of NaCl (5 mmol·L^−1^), KCl (0.17 mmol·L^−1^), CaCl_2_ (0.33 mmol·L^−1^), and MgSO_4_ (0.33 mmol·L^−1^), and incubated at 28 °C until experimental use. Embryos were harvested at 0 h post-fertilization (hpf) and maintained under standard conditions until reaching 96 hpf.

#### 2.4.2. Experimental Design In Vivo

Zebrafish larvae (96 hpf) displaying normal morphology and spontaneous swimming were selected and distributed individually into 96-well plates (Nest Biotechnology, Rahway, NJ, USA), with 10 larvae assigned to each experimental group. In the short-term exposure, larvae were exposed for 2 h to SNP at concentrations of 0.1, 0.5, 1, or 2 mmol·L^−1^ prepared in E3 medium (final volume 0.1 mL per well). After this initial treatment, the solutions were replaced with fresh E3 medium, and the larvae were maintained for an additional 22 h at 28 °C. In the long-term exposure, larvae were first pre-incubated in E3 medium for 4 h and subsequently exposed to SNP at the same concentrations for 20 h (final volume 0.1 mL per well). Control groups, consisting of untreated larvae, were incubated under identical conditions in parallel with both treatment protocols.

#### 2.4.3. Metabolic Activity

Larval media from each experimental condition were supplemented with resazurin at a final concentration of 80 μmol·L^−1^ [29]. After 24 h incubation period, corresponding to 5 days post-fertilization (dpf), the reduction in resazurin to resorufin was assessed by fluorescence measurement using a microplate reader (BioTek Synergy HT, Santa Clara, CA, USA), with excitation and emission wavelengths set at 530 and 590 nm, respectively.

#### 2.4.4. ROS Quantification

Larvae from each experimental group were rinsed with fresh E3 medium and subsequently exposed to 1 mmol·L^−1^ H_2_DCF-DA (Sigma–Aldrich^®^, St. Louis, MO, USA) in a final volume of 0.1 mL per well. Plates were incubated at 28 °C for 2 h in the dark. After incubation, fluorescence intensity was measured with a multimode microplate reader (BioTek Synergy HT, Santa Clara, CA, USA) using excitation and emission wavelengths of 480 and 530 nm, respectively. Results were normalized to the control group and expressed as percentages, represented graphically as box-and-whisker plots of three independent experiments.

#### 2.4.5. Behavior Analysis

Larval locomotor behavior was evaluated at 120 hpf to assess swimming activity, as previously described [30]. Treated and control larvae (*n* = 6 per group) were individually placed in 96-well plates containing 100 μL of E3 medium per well. After a 15-min acclimatization period, video recordings were acquired using a custom-built tracking setup. Each larva was monitored for 120 s, and locomotor parameters were analyzed using ImageJ2 and Fiji software (version 2.9.0; NIH, USA) [31,32]. Total distance traveled was defined as the cumulative displacement during the recording period, whereas mean swimming speed was calculated by dividing the total distance by the acquisition time.

### 2.5. Statistical Analyses

Statistical comparisons among experimental groups were performed using one-way analysis of variance (ANOVA). When appropriate, Tukey’s multiple comparison test was applied for comparisons across groups, and Dunnett’s test was used when experimental groups were compared with a single control. For behavioral parameters in zebrafish, repeated-measures ANOVA was employed, followed by Tukey’s post hoc test. Student’s *t*-test was used for specific pairwise comparisons. Spearman’s correlation coefficient (ρ, range −1 to +1) was calculated to investigate the associations among oxidative stress, metabolic activity, membrane integrity, and locomotor parameters. Correlation heatmaps were generated to visualize pairwise correlations, and linear regression was applied when necessary to further explore the predictive contribution of selected variables. All statistical procedures were carried out using GraphPad Prism (version 8.0; GraphPad Software, La Jolla, CA, USA) and JASP (version 0.16.2; JASP Team, Amsterdam, The Netherlands). Statistical significance was defined as *p* < 0.05.

## 3. Results

Figure 1 illustrates the experimental designs used to evaluate the toxic effects of SNP in both the short-term and long-term exposure models, applied to two neuronal cell types (mHippoE-18 and PC12) and to zebrafish larvae. In the short-term exposure, cells or larvae were exposed to SNP for 2 h, followed by replacement with fresh medium or drug-free water and maintained for an additional 22 h before analysis. In the long-term exposure, cells or larvae were pre-incubated in culture medium or water for 4 h before SNP exposure, which was then maintained for the subsequent 20 h. Control groups (C) underwent identical handling but without SNP exposure. The timeline schemes show the treatment sequence and duration for each model, highlighting the initial treatment phase and the post-treatment or pre-treatment intervals.

### 3.1. SNP-Induced Toxicity in the Short-Term Exposure on mHippoE-18 and PC12 Cells

**Figure 2:**
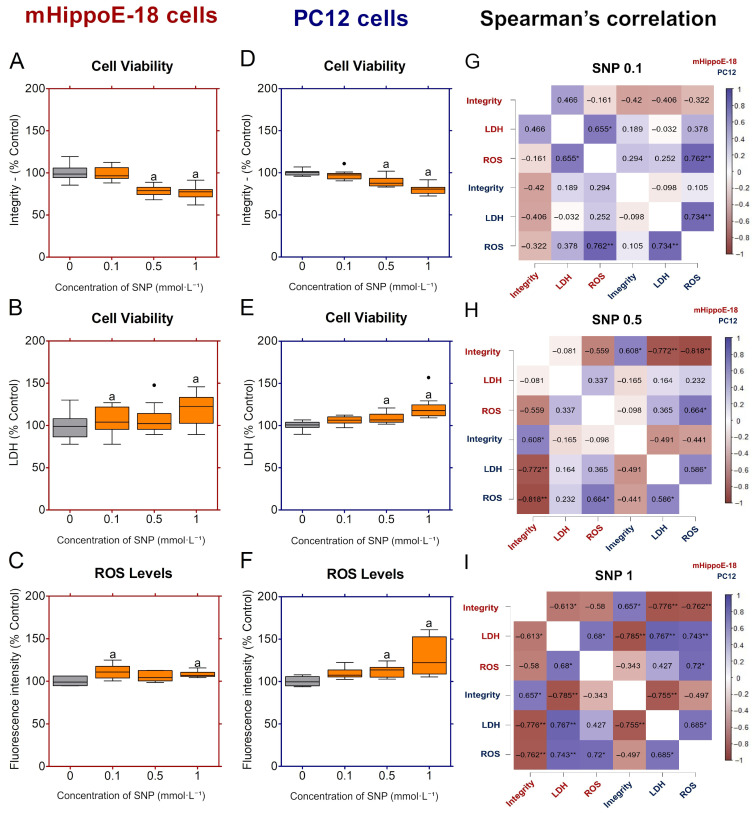
Effects of sodium nitroprusside (SNP) in the short-term exposure on cell viability, membrane integrity, and ROS production in mHippoE-18 and PC12 cells. Cells were exposed to SNP (0.1, 0.5, or 1 mmol·L^−1^) for 2 h, followed by 22 h in fresh culture medium. (**A**,**D**) Cell viability was determined by crystal violet dye (CV) assay and expressed as absorbance at 570 nm. (**B**,**E**) Membrane integrity was assessed by lactate dehydrogenase (LDH) release (U·L^−1^). (**C**,**F**) Reactive oxygen species (ROS) production was measured by H_2_DCF-DA fluorescence and expressed as percentage of control. Outlier values are represented by symbols (●) and were retained in the graphical representation to illustrate data distribution. Data are shown as box-whisker plots (minimum–maximum, median). Statistical differences compared to the control are indicated by “a” (*p* < 0.05). Spearman correlation analyses between cell viability by integrity, LDH release and ROS production in mHippoE-18 and PC12 cells exposed at 0.1, 0.5, and 1 mmol·L^−1^ SNP (**G**, **H**, and **I** respectively). The positively correlated variables are represented by the blue tone, and the negatively correlated variables are represented by the wine tone (Spearman ρ from +1 to −1). Spearman’s rank correlation analyses were conducted using JASP software, with statistical significance defined as * *p* < 0.05, and ** *p* < 0.01.

Exposure of mHippoE-18 cells to SNP resulted in a concentration-dependent decrease in cell viability (Figure 2A), and significant reductions compared to the control group were observed at 0.5 and 1 mmol·L^−1^ SNP (Figure 2A). LDH release was increased with increasing SNP concentrations (Figure 2B). ROS production was significantly elevated at 0.1 and 1 mmol·L^−1^ SNP compared to control (Figure 2C). In PC12 cells, SNP exposure did not alter CV at 0.1 mmol·L^−1^; however, a marked and significant decrease was detected at 0.5 and 1 mmol·L^−1^ (Figure 2D). LDH release remained unchanged at 0.1 mmol·L^−1^, but was significantly increased at 0.5 and 1.0 mmol·L^−1^ compared with control (Figure 2E). ROS generation increased in a concentration-dependent manner, with significant elevations observed at 0.5 and 1 mmol·L^−1^ (Figure 2F).

Spearman’s correlation analysis revealed dose-dependent and cell-type-specific association patterns between integrity, LDH release, and ROS (Figure 2G–I). At 0.1 mmol·L^−1^ SNP, correlations were generally weaker. In mHippo-E18 cells, ROS correlated positively with LDH (ρ = 0.65, * *p* < 0.05), whereas integrity showed no significant associations (Figure 2G). In PC12 cells, LDH correlated positively with ROS (ρ = 0.73, ** *p* < 0.01), while cell integrity showed no significant associations (Figure 2G). At 0.5 mmol·L^−1^ SNP, mHippo-E18 cells did not show significant correlations among viability, LDH release, and ROS levels (Figure 2H). In contrast, PC12 cells exhibited a moderate positive correlation between LDH release and ROS levels (ρ = 0.59, * *p* < 0.05), whereas viability displayed only non-significant negative correlations with both parameters. At 1 mmol·L^−1^ SNP, both cell types showed strong negative correlations between integrity and LDH (ρ ≈ −0.75, ** *p* < 0.01), consistent with membrane damage as a major determinant of reduced viability (Figure 2I). However, ROS did not correlate significantly with either integrity or LDH at this concentration, suggesting that cytotoxicity was primarily associated with membrane disruption rather than oxidative stress (Figure 2I).

### 3.2. SNP-Induced Toxicity in the Long-Term Exposure on mHippoE-18 and PC12 Cells

**Figure 3:**
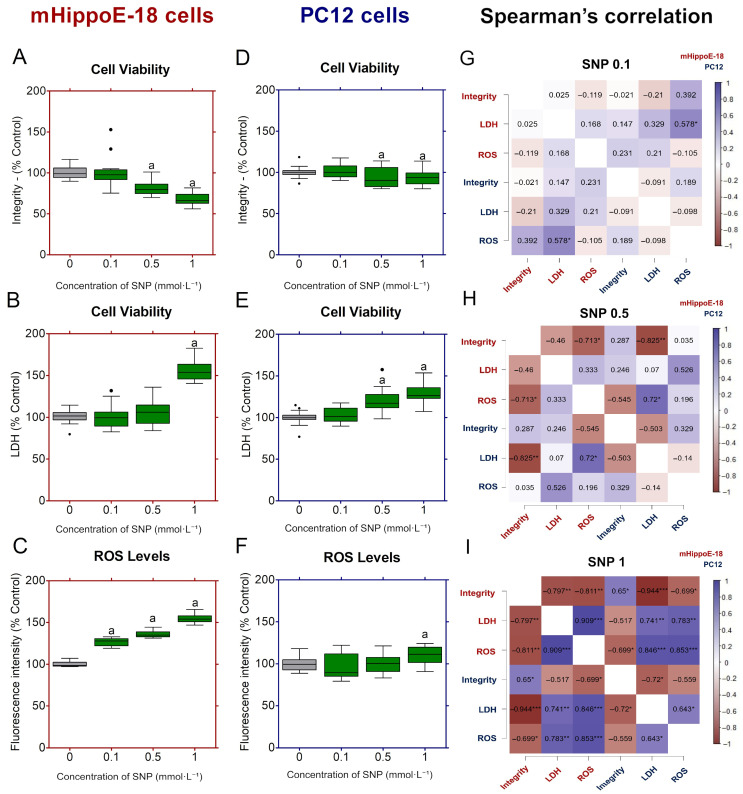
Effects of sodium nitroprusside (SNP) in the long-term exposure on cell viability, membrane integrity, and ROS production in mHippoE-18 and PC12 cells. Cells were pre-incubated in culture medium alone for 4 h, followed by exposure to SNP (0.1, 0.5, or 1 mmol·L^−1^) for 20 h. (**A**,**D**) Cell viability was determined by crystal violet dye (CV) assay and expressed as absorbance at 570 nm. (**B**,**E**) Membrane integrity was assessed by lactate dehydrogenase (LDH) release (U·L^−1^). (**C**,**F**) Reactive oxygen species (ROS) production was measured by H_2_DCF-DA fluorescence and expressed as percentage of control. Data are shown as box-whisker plots (minimum–maximum, median). Data are shown as box-whisker plots (minimum–maximum, median). Outlier values are represented by symbols (●) and were retained in the graphical representation to illustrate data distribution. Statistical differences compared to the control are indicated by “a” (*p* < 0.05). Spearman correlation analyses between cell viability by integrity, LDH release and ROS production in mHippoE-18 and PC12 cells exposed at 0.1, 0.5, and 1 mmol·L^−1^ SNP (**G**, **H**, and **I** respectively). Positive correlations are indicated by blue color gradients, whereas negative correlations are depicted using burgundy tones, with Spearman’s ρ values ranging from +1 to −1. Spearman’s rank correlation analyses were conducted using JASP software, with statistical significance defined as * *p* < 0.05, ** *p* < 0.01, *** *p* < 0.001.

In mHippo-E18 cells, SNP exposure caused a concentration-dependent reduction in cell viability, with significant decreases observed at 0.5 and 1 mmol·L^−1^ compared to control (Figure 3A). LDH release increased progressively with concentration, reaching significance at 1 mmol·L^−1^ (Figure 3B). ROS production was markedly elevated at all tested concentrations, with significant increases already detected at 0.1 mmol·L^−1^ (Figure 3C). In PC12 cells, viability was significantly reduced at 0.5 and 1 mmol·L^−1^ (Figure 3D). LDH release was significantly elevated only at 0.5 and 1 mmol·L^−1^ SNP, whereas 0.1 mmol·L^−1^ yielded values similar to control (Figure 3E). ROS generation was significantly increased at 1 mmol·L^−1^ SNP (Figure 3F).

Spearman’s correlation analysis revealed dose-dependent and cell-type-specific associations among integrity, LDH, and ROS (Figure 3G–I). At 0.1 mmol·L^−1^ SNP, neither mHippo-E18 nor PC12 cells showed significant correlations (Figure 3G). At 0.5 mmol·L^−1^ SNP, mHippo-E18 cells displayed a significant negative correlation between integrity and ROS (ρ = −0.71, * *p* < 0.05), while integrity and LDH (ρ = −0.46, n.s.) and LDH and ROS (ρ = 0.33, n.s.) were not significant (Figure 3H). By contrast, PC12 cells showed no significant correlations among any parameters (integrity vs. LDH: ρ = −0.50, n.s.; integrity vs. ROS: ρ = 0.32, n.s.; LDH vs. ROS: ρ = −0.14, n.s.) (Figure 3H). At 1 mmol·L^−1^ SNP, mHippo-E18 cells showed strong negative correlations between integrity and both LDH (ρ = −0.80, ** *p* < 0.01) and ROS (ρ = −0.81, ** *p* < 0.01), while LDH correlated positively with ROS (ρ = 0.91, *** *p* < 0.001) (Figure 3I). In contrast, PC12 cells exhibited a negative correlation between integrity and LDH (ρ = −0.72, * *p* < 0.05) and a positive correlation between LDH and ROS (ρ = 0.64, * *p* < 0.05), whereas integrity and ROS were not significantly related (ρ = −0.56, n.s.) (Figure 3I).

### 3.3. SNP-Induced Toxicity in the Short-Term Exposure on Zebrafish Larvae

**Figure 4:**
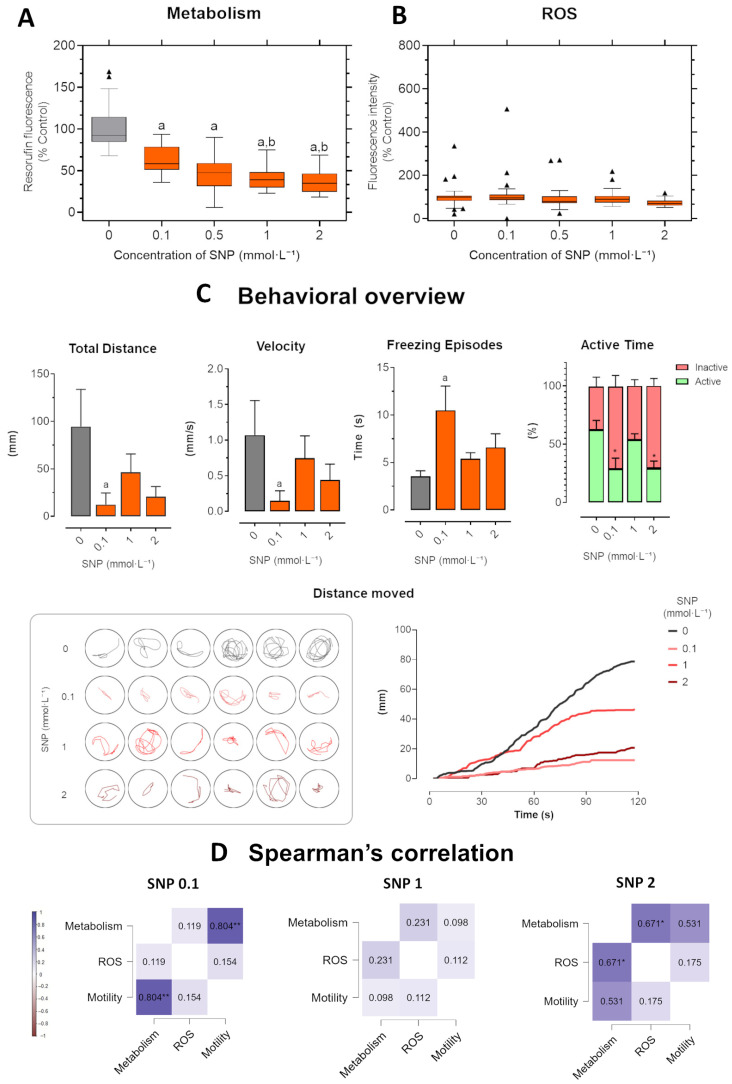
Short-term exposure in zebrafish larvae exposed to sodium nitroprusside (SNP) at concentrations of 0.1, 1, and 2 mmol·L^−1^ compared to control larvae. (**A**) Metabolic activity measured by resorufin fluorescence. (**B**) ROS production expressed as fluorescence intensity relative to control (%). Outlier values are represented by triangles (▲) and were retained in the graphical representation to illustrate data distribution. (**C**) Behavioral parameters were assessed by total distance traveled (mm), swimming velocity (mm·s^−1^), freezing episodes (s), and percentage of active versus inactive time. Lower panels illustrate representative swimming trajectories of individual larvae within wells and cumulative distance moved over 120 s. Data were analyzed using one-way ANOVA to assess differences among groups, followed by Tukey’s multiple comparison test or Dunnett’s test when comparing experimental groups with a single control (mean ± SEM; *n* = 6). Data are presented as box-and-whisker plots (minimum–maximum, median; *n* = 30) or bar graphs. Different letters indicate significant differences (*p* < 0.05): “a” vs. control group (C), “b” vs. 0.1 mmol·L^−1^ SNP, “*” between experimental groups. (**D**) Spearman’s correlation analysis of metabolism, ROS production, and motility (total distance traveled) in zebrafish larvae exposed to different concentrations of SNP. Positive correlations are shown in blue and negative correlations in burgundy (ρ range: –1 to +1). Correlation matrices were generated using JASP software (version 0.16.2; JASP Team, Amsterdam, The Netherlands). Statistical significance was set at * *p* < 0.05, and ** *p* < 0.01.

In the short-term exposure (Figure 4), zebrafish larvae exposed to SNP exhibited a dose-dependent decline in metabolic activity, measured by resorufin fluorescence. Significant reductions were detected from 0.1 mmol·L^−1^ onward, with the strongest suppression observed at 1 and 2 mmol·L^−1^ compared to both control and the 0.1 mmol·L^−1^ group (*p* = 0.005 and *p* = 0.007, respectively). ROS levels remained largely stable across concentrations, with no consistent significant differences, suggesting limited contribution of oxidative imbalance under these conditions.

SNP exposure also led to pronounced behavioral impairments compared to control larvae (Figure 4C). The total distance traveled was significantly reduced in the 0.1 mmol·L^−1^ group, while 1 and 2 mmol·L^−1^ groups showed lower values that did not reach significance. Swimming velocity followed a similar pattern, with a significant reduction at 0.1 mmol·L^−1^ and intermediate values at higher concentrations that were not statistically different from control. Freezing behavior was significantly increased at 0.1 mmol·L^−1^, whereas groups treated with 1 and 2 mmol·L^−1^ showed a non-significant tendency toward longer immobility. In contrast, the percentage of active time was markedly decreased at 0.1 and 2 mmol·L^−1^, accompanied by increased inactive time, suggesting impaired swimming ability at higher SNP concentrations.

Representative swimming trajectories confirmed these behavioral effects. Control larvae displayed longer and more exploratory movements, whereas SNP-treated groups exhibited shorter paths with reduced complexity. These differences were most evident at 0.1 mmol·L^−1^, where freezing behavior predominated. Cumulative distance plots over 120 s further demonstrated a progressive reduction in swimming activity across concentrations, with control larvae steadily increasing distance moved over time, while SNP-treated larvae showed flattened slopes, culminating in the lowest cumulative distance at 2 mmol·L^−1^.

Spearman’s rank correlation analysis revealed that associations among metabolism, ROS, and motility were generally weak and inconsistent across concentrations (Figure 4D). At 2 mmol·L^−1^, metabolism correlated positively with ROS (ρ = 0.67, * *p* < 0.05), but the association with motility was modest (ρ ≈ 0.53, n.s.). At 1 mmol·L^−1^, no significant correlations were detected among endpoints, indicating a lack of alignment between metabolic impairment, oxidative stress, and behavior. At 0.1 mmol·L^−1^, metabolism correlated strongly with motility (ρ = 0.80, ** *p* < 0.01), while no meaningful association was observed with ROS (ρ ≈ 0.12). Across concentrations, metabolism displayed very strong positive correlations (ρ = 0.87–0.99, *** *p* < 0.001), confirming a consistent dose-dependent effect of SNP. However, the absence of robust within-dose associations suggests that metabolic disruption occurred largely independently of ROS levels and behavioral outcomes in this paradigm.

### 3.4. SNP-Induced Toxicity in the Long-Term Exposure

**Figure 5:**
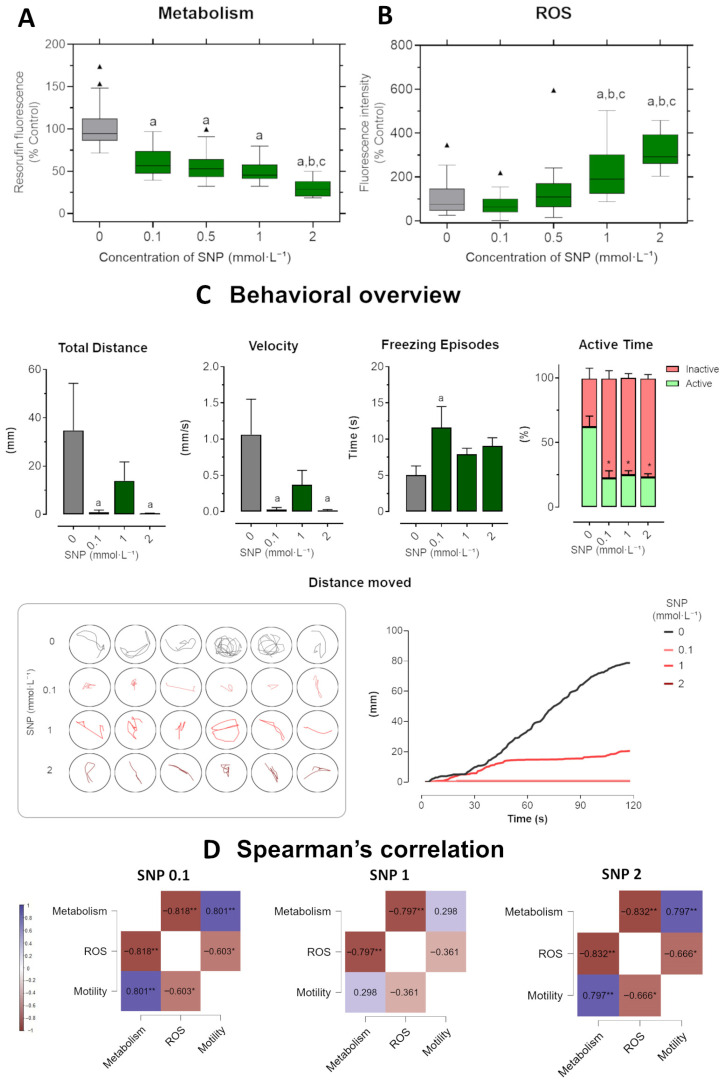
Long-term exposure in zebrafish larvae exposed to sodium nitroprusside (SNP) at concentrations of 0.1, 1, and 2 mmol·L^−1^ compared to control larvae. (**A**) Metabolic activity measured by resorufin fluorescence. (**B**) ROS production expressed as fluorescence intensity relative to control (%). Outlier values are represented by triangles (▲) and were retained in the graphical representation to illustrate data distribution. (**C**) Behavioral parameters were assessed by total distance traveled (mm), swimming velocity (mm·s^−1^), freezing episodes (s), and percentage of active versus inactive time. Lower panels illustrate representative swimming trajectories of individual larvae within wells and cumulative distance moved over 120 s. Data were analyzed using one-way ANOVA to assess differences among groups, followed by Tukey’s multiple comparison test or Dunnett’s test when comparing experimental groups with a single control (mean ± SEM; *n* = 6). Data are presented as box-and-whisker plots (minimum–maximum, median; *n* = 30) or bar graphs. Different letters indicate significant differences (*p* < 0.05): “a” vs. control group (C), “b” vs. 0.1 mmol·L^−1^ SNP, “*” between experimental groups. (**D**) Spearman’s correlation analysis of metabolism, ROS production, and motility (total distance traveled) in zebrafish larvae exposed to different concentrations of SNP. Positive correlations are shown in blue and negative correlations in burgundy (ρ range: –1 to +1). Correlation matrices were generated using JASP software (version 0.16.2; JASP Team, Amsterdam, The Netherlands). Statistical significance was set at * *p* < 0.05, and ** *p* < 0.01.

In the long-term exposure (Figure 5), zebrafish larvae pre-incubated in medium before SNP treatment showed a clear dose-dependent suppression of metabolic activity. Reductions were evident from 0.1 mmol·L^−1^, with the strongest decrease at 2 mmol·L^−1^ (*p* = 0.001), which also differed significantly from the 0.1 (*p* < 0.0001) and 0.5 mmol·L^−1^ groups (Figure 5A). ROS production was more prominently elevated than in the short-term exposure, with significant increases at 1 and 2 mmol·L^−1^ compared to control (*p* = 0.002 and *p* < 0.0001, respectively), as well as to 0.1 and 0.5 mmol·L^−1^ groups (all *p* < 0.01; Figure 5B).

SNP exposure consistently impaired locomotor activity across all tested concentrations (Figure 5C). The total distance traveled was significantly reduced at 0.1 and 2 mmol·L^−1^, with larvae showing very limited displacement throughout the assay. Swimming velocity followed the same pattern, being markedly reduced at all doses, with significant differences from control already apparent at 0.1 mmol·L^−1^. Freezing behavior was significantly increased at 0.1 mmol·L^−1^, whereas larvae exposed to 1 and 2 mmol·L^−1^ also showed longer immobility times, although these changes did not reach significance. Active time was strongly decreased across concentrations, accompanied by corresponding increases in inactive time, indicating severe locomotor suppression. Representative swimming trajectories confirmed these effects: control larvae exhibited exploratory, complex paths, while SNP-treated larvae showed minimal, restricted movements, especially at 1 and 2 mmol·L^−1^. Cumulative distance curves further emphasized this pattern, with control larvae steadily increasing distance moved over 120 s, while treated groups displayed flat slopes, culminating in near-complete inactivity at 2 mmol·L^−1^.

Spearman’s rank correlation analysis revealed robust and monotonic associations among metabolism, ROS, and motility across SNP concentrations (Figure 5D). Metabolism correlated negatively with ROS at all concentrations, indicating that increasing oxidative stress was consistently associated with impaired energy metabolism. Positive correlations between metabolism and motility were observed at 0.1 and 2 mmol·L^−1^ (ρ = 0.80, *p* < 0.01; ρ = 0.79, *p* < 0.01, respectively), suggesting that locomotor performance depends on energy availability. Conversely, ROS correlated negatively with motility at 0.1 and 2 mmol·L^−1^ (ρ = −0.60, *p* < 0.05; ρ = −0.66, *p* < 0.05, respectively), reinforcing the link between oxidative imbalance and behavioral impairment at subtoxic and higher doses.

## 4. Discussion

In this study, SNP was used as a xenobiotic NO donor to induce controlled oxidative and nitrosative stress across cellular and organismal systems, enabling a comparative assessment of toxicodynamic responses under short-term and long-term exposure paradigms. Together, the findings show that SNP elicits concentration- and time-dependent cytotoxicity detectable at the levels of metabolic activity, membrane integrity, ROS production, and behavior. The magnitude and pattern of these effects depend on cell type, exposure paradigm, and biological level of organization. These results support the view that oxidative and nitrosative stress act as early, interconnected drivers of neurodegenerative cascades rather than mere byproducts of neuronal loss, and that integrated cellular plus zebrafish models provide a relevant experimental platform for neurodegenerative drug discovery. Within this framework, the concentration range of SNP employed (0.1–2 mmol·L^−1^) was intentionally selected to generate a robust and graded nitrosative challenge, rather than to mimic physiological NO signaling. While lower micromolar concentrations of NO donors are commonly used to investigate NO–cGMP-dependent signaling pathways, higher concentrations are typically required to disrupt mitochondrial respiration and induce oxidative and nitrosative stress leading to cytotoxicity [33]. The use of millimolar concentrations therefore aligns with the objective of establishing a reproducible xenobiotic stress model capable of eliciting measurable metabolic, membrane, and behavioral phenotypes [34]. Nonetheless, we acknowledge that mixed mechanisms may contribute at higher doses, and that extrapolation to physiological NO signaling should be made with caution. Recent work has reinforced that excess ROS and RNS contribute to mitochondrial dysfunction, protein oxidation, lipid peroxidation, and DNA damage in several neurodegenerative conditions, including Alzheimer’s disease, Parkinson’s disease, amyotrophic lateral sclerosis, and related disorders [35].

SNP is widely used as an experimental NO donor, but its effects reflect a complex interplay between NO release, peroxynitrite formation, mitochondrial dysfunction, and potential cyanide contribution. NO can reversibly inhibit cytochrome c oxidase, modulate mitochondrial membrane potential, and shift the balance between survival and cell death pathways depending on local concentration and redox context [36]. In the present work, both mHippoE-18 and PC12 cells showed concentration and time-dependent reductions in metabolic activity and increases in LDH release when exposed to SNP in the short-term exposure, consistent with mitochondrial compromise and loss of membrane integrity. The stronger susceptibility of PC12 cells relative to mHippoE-18 cells is in line with previous evidence that chronic catecholaminergic or neuron-like lines often display pronounced vulnerability to nitrosative and oxidative stressors, and suggests cell type specific differences in antioxidant capacity, mitochondrial reserve, or NO signaling.

Consistent with the concentration- and time-dependent reductions in metabolic activity and increases in LDH release observed here, previous studies have demonstrated that SNP can trigger both apoptotic and non-apoptotic death in PC12 cells through established NO- and redox-mediated cytotoxic pathways. In primary cortical neurons and differentiated PC12 cells, SNP evokes robust LDH release and morphological alterations that are compatible with necrotic or late apoptotic damage, with the pattern of cell death depending on both the NO donor and the cellular phenotype [37]. In PC12 cells, alternative execution pathways beyond classical caspase-dependent apoptosis have also been reported, which may contribute to the pronounced membrane damage observed under nitrosative stress. Consistent with this interpretation, pharmacological interventions that reinforce antioxidant defenses or stress-response systems can attenuate SNP toxicity in PC12 cells. For example, a huperzine A derivative (M3) protects PC12 cells from SNP induced apoptosis by decreasing ROS and malondialdehyde levels and upregulating Hsp70, suggesting that reinforcement of redox buffering and chaperone systems can counteract NO mediated injury [38,39]. The tight association between SNP concentration, metabolic suppression, and LDH release observed in both cell lines in the present study is therefore coherent with this literature and supports the relevance of these in vitro models for capturing key components of NO-induced neuronal damage.

The differential patterns observed between the short-term and long-term exposure paradigms in both cell lines indicate that the timing of SNP exposure critically shapes the toxic response. In the short-term exposure, cells are challenged with SNP after attachment and adaptation, which may better mimic an acute insult to a relatively stable network. In the long-term exposure, pre-exposure to SNP occurs before or during early cell adaptation, which could theoretically induce a preconditioning-like response, but in practice still results in reduced metabolic activity and increased LDH release at higher concentrations. The observation that Spearman correlations between metabolic activity, LDH, and ROS remain strong in both paradigms suggests that, despite differences in exposure sequence, SNP primarily disrupts cell viability through convergent oxidative and nitrosative pathways. However, subtle differences in correlation patterns between mHippoE-18 and PC12 cells indicate that the thresholds for ROS accumulation and loss of viability are not identical and should be considered when choosing cell systems for neuroprotective screening.

At the in vivo level, the concentration-dependent impairments in locomotor behavior observed in zebrafish larvae provide a complementary functional perspective by integrating systemic physiology, intact neural circuits, and behavior [40,41,42,43]. In the short-term exposure paradigm, SNP produced modest reductions in metabolic activity at the highest concentrations, limited changes in whole-larval ROS levels, but clear and reproducible deficits in locomotor performance. Total distance traveled, swimming trajectories, and activity profiles declined progressively with increasing SNP concentration, indicating that behavioral endpoints represent more sensitive functional readouts than bulk metabolic or redox measures under these conditions. This dissociation suggests that SNP perturbs neuronal function and network dynamics at concentrations where localized oxidative or nitrosative stress in specific neuronal populations may not be captured by whole-organism ROS measurements. The non-linear behavioral responses observed in this paradigm are consistent with reports of non-monotonic dose–response patterns for NO donors, including SNP, and likely reflect the dual role of NO and related RNS in redox signaling and cytotoxicity, where adaptive or modulatory processes may prevail at lower concentrations before mitochondrial and redox homeostasis are disrupted at higher doses [44,45]. Although altered locomotor behavior is widely used as a functional readout of neurotoxicity in zebrafish larvae, it should be interpreted as an integrated phenotype rather than a direct measure of neuronal cell loss. Alternative contributors, including impaired cardiac performance, muscle function, or nonspecific physiological distress, cannot be fully excluded under the present experimental conditions. However, the concentration-dependent and reproducible nature of the behavioral changes, together with their association with metabolic suppression and redox imbalance, supports a primary involvement of neural network dysfunction at early stages of toxic stress. Structural or cell-type-specific analyses were beyond the scope of this study and will be required to further resolve the relative contribution of neural versus non-neural targets. In addition to redox-mediated mechanisms, NO may also engage soluble guanylate cyclase (sGC) and cGMP-dependent signaling pathways that modulate neuronal excitability, synaptic transmission, and locomotor behavior. Although this pathway was not assessed experimentally here, it may contribute to the behavioral phenotypes observed in zebrafish and warrants investigation in future studies [33].

In the long-term exposure zebrafish paradigm, pre-incubation with SNP amplified metabolic suppression and ROS production and resulted in more severe and uniform locomotor deficits. At the highest concentration, larvae exhibited near-complete inactivity, indicating a loss of functional reserve under sustained nitrosative stress. Compared with the short-term exposure condition, behavioral and biochemical responses followed a more monotonic dose–response pattern, with strong correlations between SNP concentration, ROS levels, and locomotor parameters. These features indicate that exposure sequence critically shapes the toxicodynamic profile of SNP in vivo and that prolonged exposure leads to exhaustion of compensatory mechanisms that may partially buffer acute stress. From a translational perspective, the long-term exposure model provides a robust framework for defining concentration ranges suitable for phenotypic screening, where partial rescue of behavior or redox balance can be quantitatively detected before floor effects dominate.

The hypokinesia, reduced activity profiles, and altered swimming trajectories observed here are consistent with recent zebrafish studies showing that locomotor readouts are highly sensitive to early neurodegenerative processes and can be combined with molecular profiling to dissect underlying mechanisms. For example, zebrafish larvae exposed to the mitochondrial complex I inhibitor 1-Methyl-4-Phenylpyridinium (MPP^+^) display pronounced hypokinesia, loss of light evoked responses, and broad proteomic alterations in pathways related to mitochondrial metabolism, redox regulation, proteostasis, and synaptic organization, effectively modeling early Parkinsonian-like dysfunction [30]. The current study extends this concept to a NO donor, showing that SNP can also generate robust locomotor and metabolic phenotypes in zebrafish larvae, with patterns that resemble early stage neurotoxic stress rather than advanced structural degeneration. This reinforces the suitability of zebrafish larvae as an in vivo platform for probing early events that are likely to be more amenable to therapeutic modulation.

Mechanistically, the combined cellular and zebrafish results support a model in which NO released from SNP interferes with mitochondrial respiration, generates peroxynitrite through reactions with superoxide, enhances ROS generation, and perturbs redox-sensitive signaling pathways, ultimately contributing to nitrosative stress [46,47]. At low to moderate SNP concentrations, cells and larvae may activate compensatory responses, including antioxidant enzyme upregulation, stress protein induction, and transient metabolic adjustments, which can preserve viability despite rising nitrosative pressure. At higher concentrations, these defenses are overwhelmed, resulting in significant loss of metabolic activity, increased LDH release, elevated ROS and, in zebrafish, severe locomotor impairment. The observation that PC12 cells and zebrafish larvae exhibit particularly pronounced functional deficits suggests that systems with high energy demand and complex network activity are especially vulnerable to sustained NO driven oxidative stress, consistent with reports of mitochondrial bioenergetic disruption and redox-dependent neuronal vulnerability [33,34].

From the standpoint of neurodegenerative drug discovery, the use of short-term and long-term exposure paradigms across cellular and zebrafish models provides several advantages [6]. First, it allows the distinction between compounds that mitigate damage when administered after the onset of stress, as modeled by short-term exposure, and those that prevent or attenuate injury when present before or during exposure, as captured by the long-term exposure paradigm. Second, it enables cross validation of candidate neuroprotective effects at multiple biological levels, where improved cell viability and reduced ROS in vitro can be aligned with preserved locomotor behavior in vivo. Third, the zebrafish platform facilitates high-throughput phenotypic screening [5] under conditions that recapitulate early neurodegenerative processes, where subtle functional improvements can be quantified with automated tracking systems. Finally, the shared reliance on a single NO donor across models simplifies interpretation of structure–activity relationships for new molecules targeting redox imbalance, mitochondrial pathways, or NO signaling, a concept supported by foundational insights into mitochondrial dysfunction and oxidative stress in neurodegeneration [1,48]. By integrating neuronal cell lines and zebrafish larvae, the present work positions SNP as a versatile xenobiotic model for probing early redox-driven neurotoxicity and for supporting translational screening of compounds with neuroprotective or therapeutic potential.

Some limitations should be considered when interpreting these data. SNP releases not only NO but also cyanide and iron, and the relative contribution of each species to toxicity can vary with concentration, exposure time, and tissue context. Although the present study was not designed to determine the specific contribution of NO, cyanide, or iron to the cellular and behavioral phenotypes observed, we agree that additional mechanistic approaches would be valuable to clarify these relationships. In this regard, future studies employing NO scavengers, cyanide-neutralizing agents, or iron chelators may help distinguish nitrosative stress-driven injury from mitochondrial impairment caused by cyanide. Such strategies would enable the dissociation of overlapping toxicodynamic pathways and refine the mechanistic interpretation of SNP as a xenobiotic model. Whole larval ROS measurements may underestimate localized oxidative and nitrosative stress in discrete neuronal populations, and complementary approaches such as region-specific redox imaging or targeted biomarkers would refine mechanistic interpretations. It should also be noted that H_2_DCF-DA predominantly detects peroxide-type ROS and does not selectively capture reactive nitrogen species (RNS), which may contribute to behavioral alterations under conditions where bulk ROS levels remain unchanged. In addition, the in vitro models used here do not fully capture the complexity of mature neural circuits, glial contributions, or neuroimmune interactions that modulate NO signaling in the mammalian brain. Despite these limitations, the convergence observed between cell-based and zebrafish endpoints supports the translational relevance of the present approach.

## 5. Conclusions

The data indicate that SNP induces coherent patterns of oxidative and nitrosative stress across mHippoE-18 cells, PC12 cells, and zebrafish larvae in both short-term and long-term exposure paradigms. Within this framework, SNP is reinforced as a xenobiotic NO donor suitable for comparative neurotoxicity profiling across cellular and organismal models. Metabolic impairment, loss of membrane integrity, increased ROS, and locomotor deficits emerge in a dose- and time-dependent manner, with PC12 cells and zebrafish behavior showing particular sensitivity. These findings position the combined cellular plus zebrafish SNP models as a versatile framework for identifying and characterizing candidate compounds with neuroprotective or therapeutic potential that target early redox-driven events in neurodegeneration and support their use in future studies integrating behavioral-, biochemical-, and omics-level readouts.

## Data Availability

The original contributions presented in this study are included in the article/Supplementary Material. Further inquiries can be directed to the corresponding author.

## Supplementary Materials

The following supporting information can be downloaded at: https://www.mdpi.com/article/10.3390/jox16010029/s1, Figure S1: Representative image of mHippoE-18 cells obtained by light microscopy (Axioskop 2, Zeiss, Germany). Magnification: 100×; Figure S2: Representative image of PC12 cells obtained by light microscopy (Axioskop 2, Zeiss, Germany). Magnification: 100×; Figure S3: Representative stereomicroscopy image of zebrafish larvae (96 hpf) acquired with an LED2500 stereomicroscope (Leica Microsystems, Wetzlar, Germany). Magnification: 25×.

## Abbreviations

The following abbreviations are used in this manuscript:

ANOVA	Analysis of Variance
ATCC	American Type Culture Collection
BBB	Blood–Brain Barrier
CEUA	Ethics Committee on the Use of Animals
CV	Crystal Violet
DMEM	Dulbecco’s Modified Eagle Medium
dpf	Days Post-Fertilization
E3	Embryo Medium
EDTA	Ethylenediaminetetraacetic Acid
FBS	Fetal Bovine Serum
hpf	Hours Post-Fertilization
H_2_DCF-DA	2′,7′-Dichlorodihydrofluorescein Diacetate
H_2_O_2_	Hydrogen Peroxide
LDH	Lactate Dehydrogenase
MPP^+^	1-Methyl-4-Phenylpyridinium
NADH	Nicotinamide Adenine Dinucleotide (Reduced Form)
NO	Nitric Oxide
PC12	Rat Pheochromocytoma Cell Line
PBS	Phosphate-Buffered Saline
ROS	Reactive Oxygen Species
RNS	Reactive Nitrogen Species
SEM	Standard Error of the Mean
SNP	Sodium Nitroprusside
WT	Wild-Type
