# Supplementary material for: Sodium Nitroprusside as a Xenobiotic Model of Oxidative and Nitrosative Stress in Cellular and Zebrafish Systems
Source: J Xenobiot. 2026 Feb 6;16(1):29. doi: 10.3390/jox16010029 (PMC12921788; doi:10.3390/jox16010029)

Supplementary Materials: Sodium Nitroprusside as a Xenobiotic Model of Oxidative and Nitrosative Stress in Cellular and Zebrafish Systems

Carlos Alberto-Silva, Felipe Assumpção da Cunha e Silva, Brenda Rufino da Silva, Leticia Ribeiro de Barros, Adolfo Luis Almeida Maleski, and Maricilia Silva Costa

Figure S1: Representative image of mHippoE-18 cells obtained by light microscopy (Axioskop 2, Zeiss, Germany). Magnification: 100×.

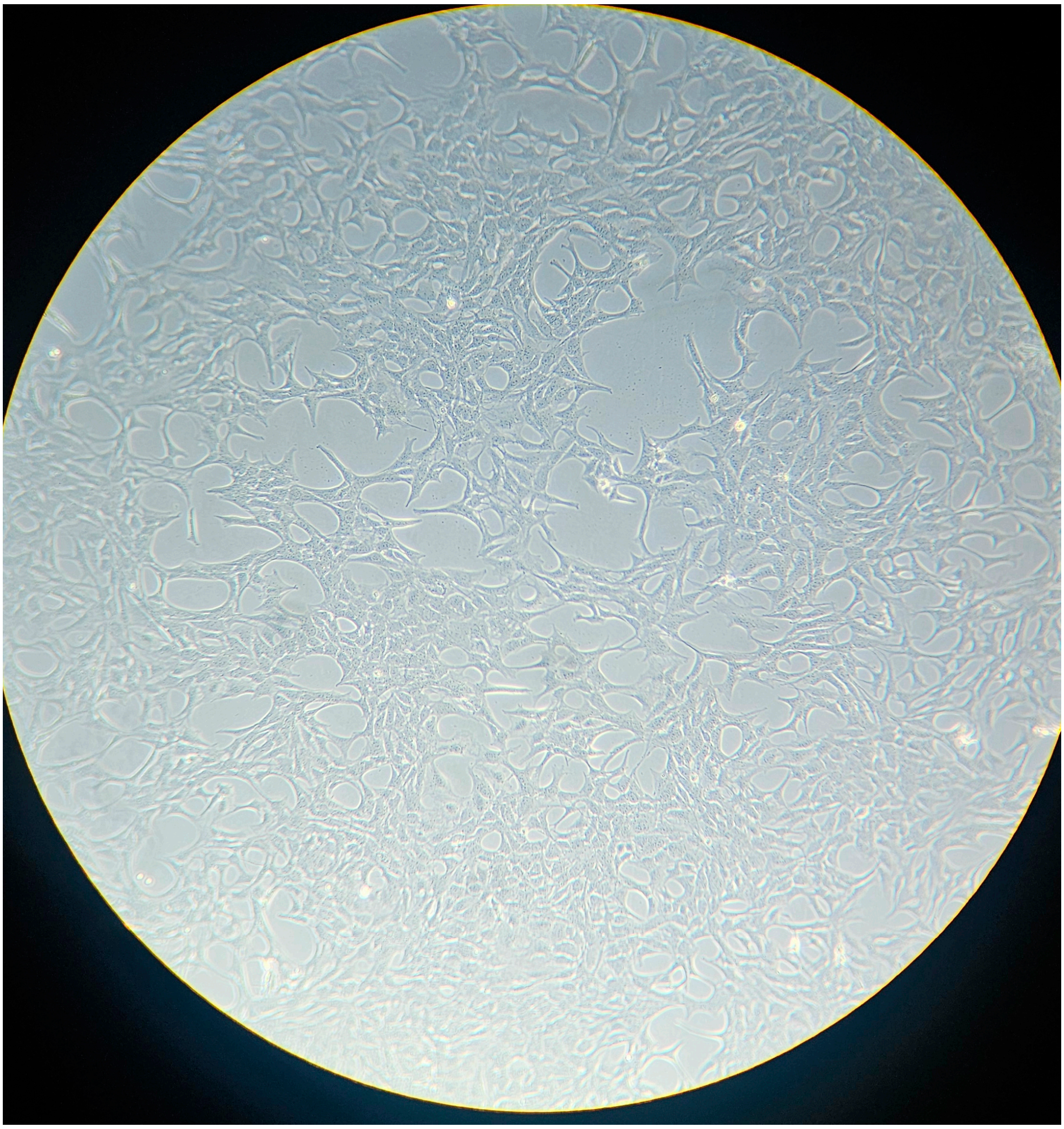

Figure S2: Representative image of PC12 cells obtained by light microscopy (Axioskop 2, Zeiss, Germany). Magnification: 100×.

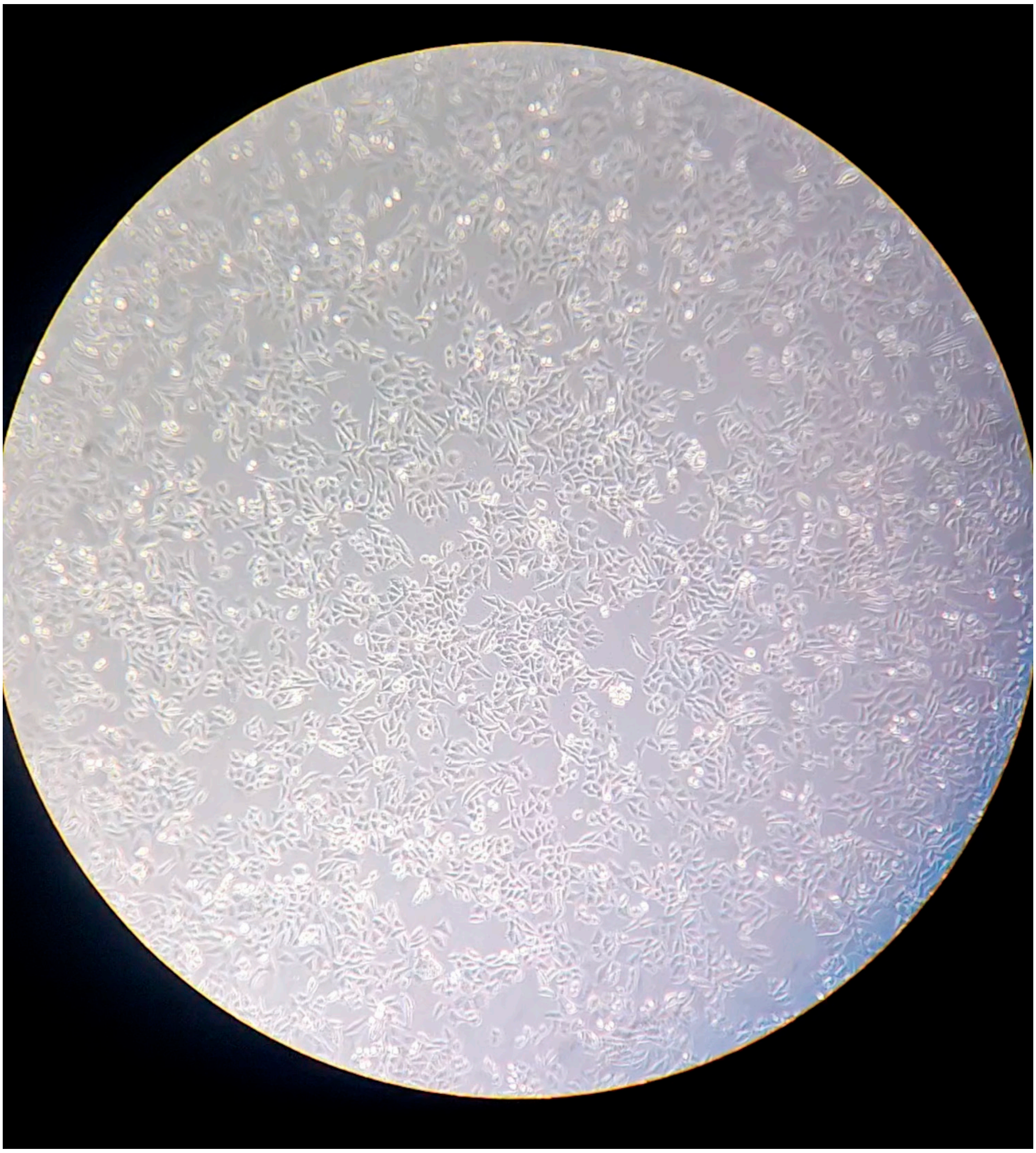

Figure S3: Representative stereomicroscopy image of zebrafish larvae (96 hpf) acquired with an LED2500 stereomicroscope (Leica Microsystems, Wetzlar, Germany). Magnification: 25×.

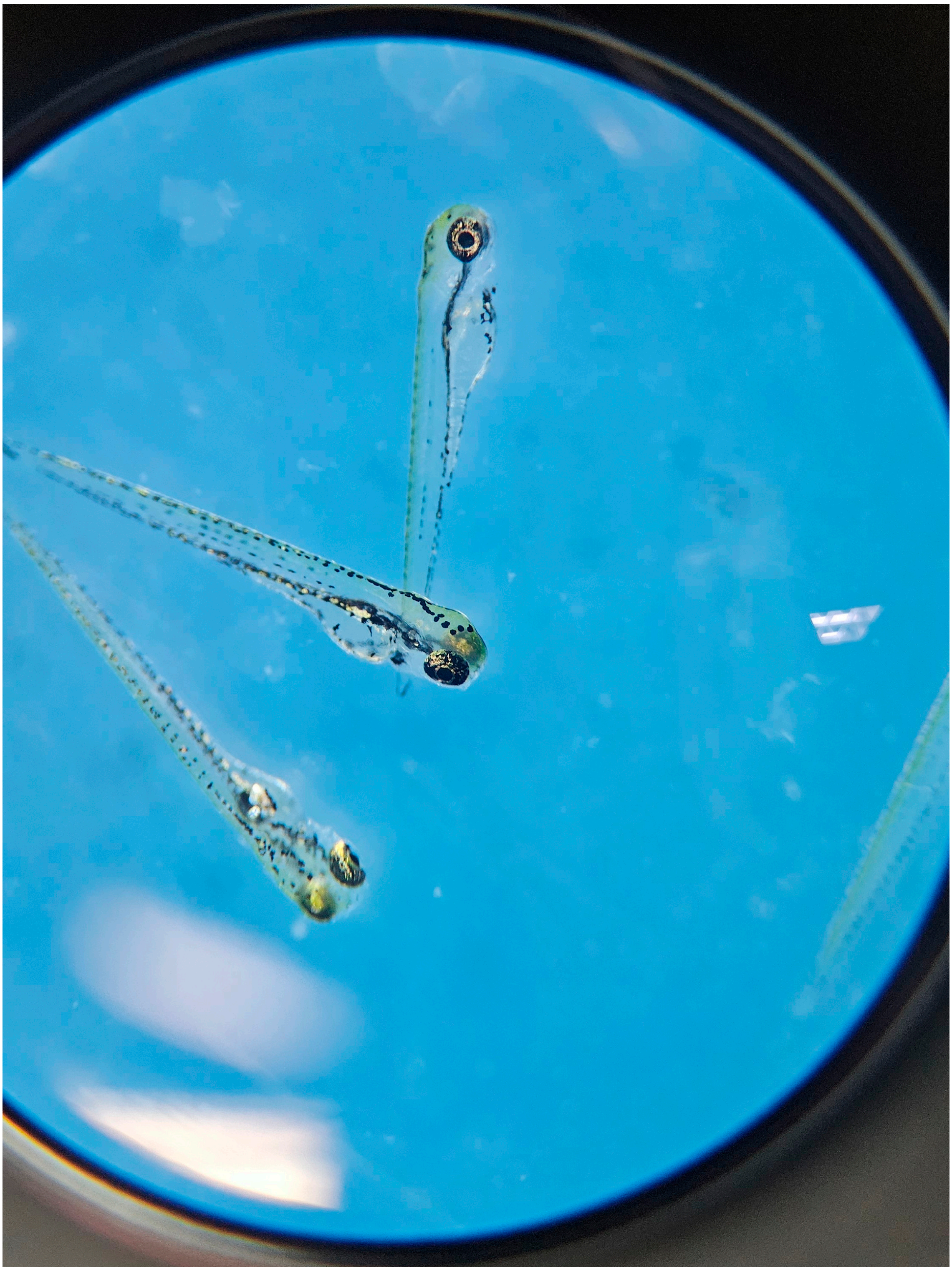

Supplement: Supplementary file 1 [file jox-16-00029-s001.zip › jox-4113764-supplementary.pdf]
